# Chronic Spontaneous Urticaria Following COVID-19 Vaccination: A Case Report and Discussion on Allergic Reactions and Vaccine Safety

**DOI:** 10.7759/cureus.44860

**Published:** 2023-09-07

**Authors:** Jesus Ivan Martinez-Ortega, Felipe de Jesus Perez Hernandez

**Affiliations:** 1 Department of Dermatology, Dermatological Institute of Jalisco "Dr. Jose Barba Rubio", Zapopan, MEX; 2 Department of Internal Medicine, Pensiones Medical Center, Merida, MEX

**Keywords:** chronic spontaneous urticaria, sars-cov-2, allergic reaction, vaccine safety, covid-19 vaccine

## Abstract

This case report examines a rare occurrence of chronic spontaneous urticaria (CSU) following the administration of the CoronaVac vaccine for COVID-19. The patient developed persistent urticarial lesions that appeared and disappeared over an extended period after receiving the vaccine. The diagnosis of CSU was supported by histopathological examination and the close temporal correlation between symptom onset and vaccination. The discussion focuses on the immune mechanisms involved in CSU, the potential triggers of allergic reactions to COVID-19 vaccines, and the importance of further research to identify specific allergenic components. This case underscores the need for vigilance in monitoring and reporting adverse events related to COVID-19 vaccination to ensure vaccine safety and optimize public health strategies.

## Introduction

Within the framework of the COVID-19 pandemic and the emergence of different types of vaccines, along with the mass vaccination of the global population, multiple adverse events have been reported, with the most common being allergic reactions and urticaria [1].

Chronic spontaneous urticaria (CSU) is defined by recurring urticaria lesions that last for a period of six weeks or more. The urticarial skin lesions typically appear and disappear within less than four hours. There have been few reported cases of CSU associated with the COVID-19 vaccine. In this context, we consider it important to communicate this type of dermatosis as an adverse effect of the various COVID-19 vaccines [1,2].

## Case presentation

A 61-year-old female with a medical history of hypertension and hypothyroidism developed a pruritic rash on the trunk and extremities few hours after receiving the second dose of CoronaVac vaccine for SARS-CoV-2. Those skin lesions had been waxing and waning off for 12 weeks, the rash used to disappear after 30-60 minutes and reappear at a different random location.

**Figure 1:**
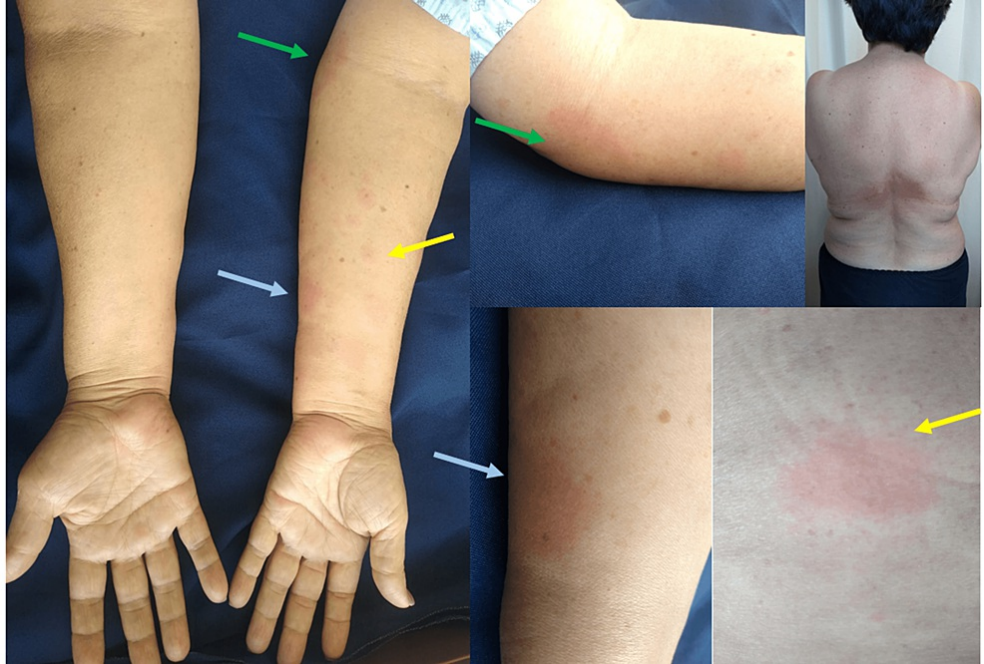
Clinical image Clinical lesions showing wheals and erythematous rash.

On physical examination, she had typical, raised urticarial lesions concentrated on her palms, upper and lower limbs, abdomen, chest and sparing her face. There was neither dermatographism nor angioedema, lung auscultation did not reveal any wheeze. There was neither lymphadenopathy nor hepatosplenomegaly (Figure 1).

**Figure 2:**
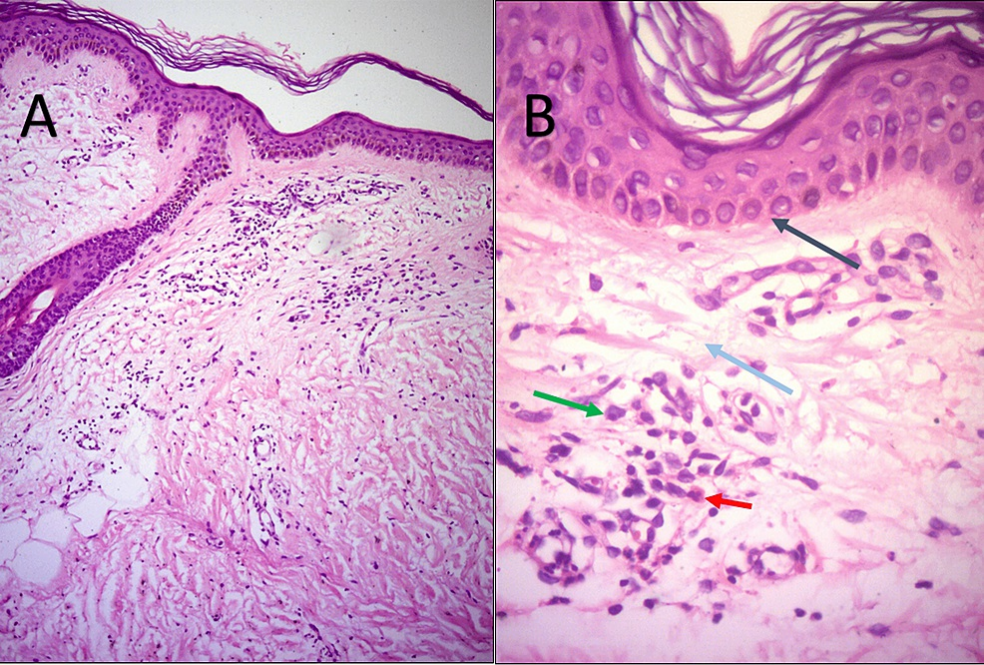
Hematoxylin & eosin stain Skin lesion biopsy histopathology (H&E stain) showing spongiosis in the epidermis and mild immune infiltration with edema within the dermis.

Blood tests were unremarkable with a normal haematological profile without eosinophilia. On the histopathology exam, there was epidermal spongiosis, and dermal interstitial edema, in addition to a mixed perivascular inflammatory infiltrate composed mainly of polymorphonuclear cells and eosinophils (Figure 2). We integrated the diagnosis of CSU presumably to CoronaVac vaccine, due to the close temporal correlation. We started antihistaminic therapy fexofenadine 180 mg daily with complete resolution two weeks later on the follow-up.

## Discussion

**Figure 3:**
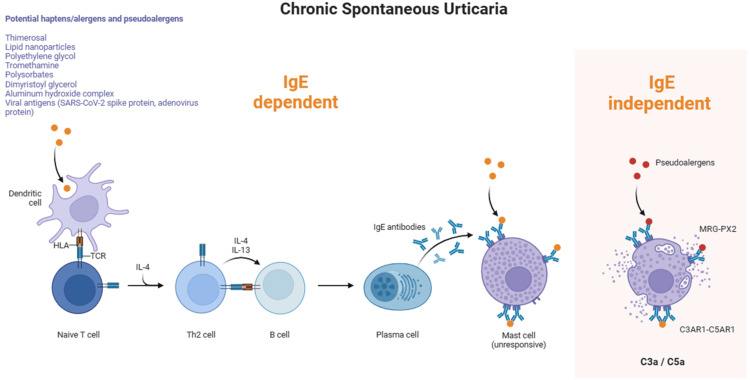
Potential components of anti-COVID-19 vaccines that can function as haptens The image showcases potential components of anti-COVID-19 vaccines that can function as haptens. These haptens can induce immune responses through different mechanisms, including the dimerization of high-affinity IgE receptors (FcεRI) and non-IgE-dependent immediate reactions. Immediate reactions can occur through direct interactions of pseudoallergens with G-protein-coupled receptors or as a result of complement activation in individuals with specific IgG antibodies against vaccine components. The image was created in Biorender and adapted from Ref. [1].

As mentioned earlier, if symptoms persist for more than six weeks, CSU should be considered, and potential triggers need to be identified. The immunological response in CSU can be either IgE-dependent or IgE-independent, with the latter being orchestrated by IgG targeting the FcεRI receptors. It is most likely that the vaccine drives an IgE-dependent immune response (Figure 3) [1].

Vaccines have a significant impact on the skin and mucosa as boundary surfaces to the environment due to the general activation of the immune system they induce [1]. Although allergic reactions to COVID-19 vaccines are rare, there have been reports of Th2-driven dermatoses such as urticaria and CSU associated with different types of vaccines, each with unique characteristics and compositions [2].

Allergic reactions to vaccines can be caused by residual non-human proteins, preservatives, or excipients that act as potential haptens. Some examples of these substances include lipid nanoparticles in mRNA vaccines, polyethylene glycols (PEGs), polysorbates, dimyristoyl glycerol, thimerosal, and tromethamine as can be seen in Figure 3 [1].

CoronaVac is an inactivated vaccine against COVID-19 that is produced using African green monkey kidney cells (Vero cells) inoculated with the SARS-CoV-2 (CN02 strain). After the incubation period, the virus is harvested, inactivated with β-propiolactone, concentrated, purified, and then absorbed onto aluminum hydroxide [3,4]. Unlike mRNA and viral vector vaccines, CoronaVac does not contain excipients such as polysorbate or PEG, which are commonly associated with allergic reactions in vaccines. The allergic components in CoronaVac are currently unknown and require further research [1].

Various vaccine components can act as haptens and trigger predominantly Th2-polarized inflammatory reactions, characterized by increased levels of interleukins IL-4 and IL-13, as well as urticarial symptoms [2]. Excipients such as PEG and polysorbate are believed to be potential causes of allergic reactions to COVID-19 vaccines [1].

The aluminum hydroxide complex used in vaccines is diluted in a solution of sodium chloride, phosphate-buffered saline, and water, sterilized, and filtered before injection. Aluminum salt is a commonly used adjuvant, and recent studies have indicated that it can induce TH2 cell-mediated immune responses [4,5].

Furthermore, it has been suggested by Triwatcharikorn et al. that allergic reactions may not be due to vaccine excipients but rather a cross-reactive immune response to the SARS-CoV-2 spike protein, possibly due to previous coronavirus infections. In other words, it may be through an IgE-independent mechanism (see Figure 3) by antibodies against the SARS-CoV-2 spike protein [5].

Regardless of the underlying cause, whether it is attributed to excipients or viral antigens, the use of adjuvants such as TH1 cell-skewing modified alum or CpG can potentially help mitigate these safety concerns. Adjuvants are substances that enhance the immune response to vaccines, and they can be engineered to promote a TH1 cell-mediated immune response, which is associated with a lower likelihood of allergic reactions compared to a TH2 response. By shifting the immune response towards a TH1 profile, the risk of allergic reactions, including chronic spontaneous urticaria, may be reduced. Further research in this area can provide valuable insights into optimizing vaccine safety and efficacy [6,7].

## Conclusions

In conclusion, this case report highlights a rare occurrence of CSU following the CoronaVac vaccine for COVID-19. Allergic reactions and urticaria are uncommon but recognized adverse events associated with COVID-19 vaccination. Further investigation is needed to determine the specific allergic components in CoronaVac and understand the underlying mechanisms. Monitoring and reporting such cases are crucial for enhancing vaccine safety and optimizing vaccination strategies worldwide.
